# OPTIA-AF trial: A randomized study of rhythm-guided antithrombotic strategy after atrial fibrillation ablation in patients with prior drug-eluting stent implantation

**DOI:** 10.1016/j.hroo.2026.02.002

**Published:** 2026-02-09

**Authors:** Dong-Hyeok Kim, Yeji Kim, Seung Woo Lee, Jungmin Kang, Junbeom Park, OPTIA-AF investigators

**Affiliations:** 1 Division of Cardiology, Ewha Womans University Seoul Hospital, Seoul, South Korea; 2 Division of Cardiology, Ewha Womans University Mokdong Hospital, Seoul, South Korea

**Keywords:** Atrial fibrillation, Catheter ablation, Noninferiority trial, Drug-eluting stent (DES), Antithrombotic therapy, Non–vitamin K antagonist oral anticoagulant (NOAC), Single antiplatelet therapy (SAPT), Rhythm-guided strategy

## Abstract

**Background:**

Recent randomized studies suggest that oral anticoagulation may be safely discontinued in carefully selected patients who maintain durable sinus rhythm (SR) after atrial fibrillation (AF) ablation. However, patients with previous drug-eluting stent (DES) implantation represent a distinct population in whom residual coronary ischemic risk persists and long-term antiplatelet therapy remains clinically relevant. The optimal antithrombotic strategy in this setting remains uncertain.

**Objective:**

The Optimal Postablation Therapy for Ischemic and Arrhythmic Risk in Atrial Fibrillation (OPTIA-AF) trial was designed to evaluate whether discontinuation of non–vitamin K antagonist oral anticoagulation (NOAC) with transition to single antiplatelet therapy is noninferior to continued NOAC therapy in patients with durable SR after AF ablation and previous DES implantation.

**Methods:**

OPTIA-AF is a prospective, multicenter, randomized controlled trial enrolling patients with nonvalvular AF who have maintained ≥12 months of documented SR after catheter ablation and are ≥12 months removed from DES implantation. Participants are randomized 1:1 to continued NOAC therapy or NOAC discontinuation with single antiplatelet therapy. Structured rhythm surveillance using electrocardiography and ambulatory monitoring is mandated throughout follow-up.

**Results:**

The primary end point is a 24-month net clinical outcome composite of ischemic stroke, systemic embolism, myocardial infarction, definite or probable stent thrombosis, cardiovascular death, and major bleeding. Key secondary end points include individual components of the composite endpoint, clinically relevant nonmajor bleeding, AF recurrence, AF burden, arrhythmia-related hospitalization, and repeat ablation. Approximately 1000 patients will be enrolled to provide adequate power for noninferiority testing.

**Conclusion:**

OPTIA-AF will provide randomized evidence to inform a rhythm-guided, individualized antithrombotic strategy after AF ablation in patients with concomitant coronary artery disease and previous DES implantation.

**Key Findings**

▪ The Optimal Postablation Therapy for Ischemic and Arrhythmic Risk in Atrial Fibrillation (OPTIA-AF) is a randomized trial evaluating a rhythm-guided antithrombotic strategy after atrial fibrillation ablation in patients with prior drug-eluting stent implantation.
▪ The trial tests whether discontinuation of non–vitamin K antagonist oral anticoagulation, with transition to single antiplatelet therapy is non-inferior to continued anticoagulation.
▪ The study integrates durable sinus rhythm, residual coronary ischemic risk, and bleeding risk to individualize long-term antithrombotic therapy.
▪ OPTIA-AF incorporates standardized rhythm surveillance and clinically meaningful composite end points, including myocardial infarction.

## Introduction

Atrial fibrillation (AF) and coronary artery disease (CAD) frequently coexist, resulting in a growing population of patients who require long-term antithrombotic therapy to mitigate competing risks of thromboembolism and coronary ischemia. Current guideline-directed management generally recommends lifelong oral anticoagulation (OAC) in patients with AF based on stroke risk stratification, whereas antiplatelet therapy remains central to the prevention of stent-related ischemic events after percutaneous coronary intervention (PCI) with drug-eluting stent (DES) implantation.^1^, ^2^, ^3^, ^4^ Although these strategies are individually evidence based, their intersection presents a persistent clinical challenge, particularly in patients who have undergone catheter ablation for AF.

Catheter ablation has become an established rhythm-control strategy, with a substantial proportion of patients achieving durable maintenance of sinus rhythm (SR). Accumulating evidence suggests that sustained SR after successful ablation is associated with a marked reduction in AF burden, atrial stasis, and prothrombotic signaling, thereby attenuating AF-related thromboembolic risk.^5^, ^6^, ^7^ On this basis, OAC discontinuation after ablation has been explored in selected patients, particularly those with low CHA_2_DS_2_-VASc scores and no additional indications for anticoagulation.^8^, ^9^, ^10^ However, most previous studies evaluating postablation anticoagulation have excluded patients with significant CAD or have not specifically addressed populations requiring ongoing antiplatelet therapy.

Although AF recurrence may be detected early after catheter ablation, rhythm status during the first several months is influenced by transient postprocedural factors and may not reliably reflect durable suppression of atrial arrhythmogenic substrate. Therefore, emerging randomized data, including ALONE-AF and OCEAN, have adopted approximately 12 months of stable SR as a clinically meaningful threshold when considering long-term modification of anticoagulation strategy. This interval represents a pragmatic balance across biological plausibility, rhythm certainty, and clinical applicability.

In patients with previous DES implantation, this dilemma is particularly pronounced. Contemporary guidelines generally recommend OAC monotherapy beyond 12 months after PCI in patients with AF to balance ischemic and bleeding risks.^11^, ^12^, ^13^ However, these recommendations implicitly assume persistent AF-related thromboembolic risk and do not adequately account for patients in whom AF recurrence has been effectively suppressed by durable SR after ablation. In such patients, residual coronary ischemic risk related to previous stent implantation may persist, creating a clinically important area of uncertainty regarding the optimal long-term antithrombotic strategy.

Importantly, thromboembolic events in AF and ischemic events after PCI arise from distinct pathophysiological mechanisms. AF-related thromboembolism is primarily driven by atrial rhythm instability and stasis, whereas late ischemic events after DES implantation are predominantly platelet mediated and related to vascular healing and local stent pathology.^14^, ^15^, ^16^ In patients who maintain sustained SR after ablation, the balance of residual risk may therefore shift away from AF-related thromboembolism toward coronary ischemic mechanisms, raising the possibility that antiplatelet therapy—rather than systemic anticoagulation—may be sufficient for long-term risk mitigation in carefully selected individuals.

Against this background, there remains a clear unmet need for randomized evidence evaluating a rhythm-guided antithrombotic strategy in patients with AF who maintain durable SR after catheter ablation and have a history of DES implantation. The Optimal Postablation Therapy for Ischemic and Arrhythmic Risk in Atrial Fibrillation (OPTIA-AF) trial was designed to address this gap by testing whether long-term antithrombotic therapy can be individualized according to objective rhythm outcomes and residual coronary risk rather than historical AF diagnosis alone.

## Objectives

Accordingly, the OPTIA-AF trial (evaluating rhythm-guided antithrombotic strategy after AF ablation in patients with previous DES implantation) was designed to assess the noninferiority of a strategy of non–vitamin K antagonist OAC (NOAC) discontinuation with transition to single antiplatelet therapy (SAPT) compared with continued NOAC therapy in terms of net clinical outcomes, while exploring whether a rhythm-guided approach may reduce bleeding risk without compromising ischemic or arrhythmic safety.

## Methods

### Study design and population

OPTIA-AF is a prospective, multicenter, open-label, randomized controlled trial designed to evaluate a rhythm-guided antithrombotic strategy in patients with AF who maintain durable SR after catheter ablation and have a history of DES implantation.

Enrollment in OPTIA-AF is performed at a predefined, uniform clinical decision point, defined as ≥12 months after the most recent AF ablation and ≥12 months after the most recent DES implantation, provided that patients have maintained documented SR during this period. This time point represents the standardized moment at which long-term antithrombotic therapy is reassessed in routine clinical practice.

Although AF ablation or PCI may have been performed earlier, patients are not randomized retrospectively. All participants are enrolled and randomized prospectively at this fixed postablation time point, ensuring consistency in the clinical context of treatment decision making across the study population. Accordingly, time 0 for all outcome analyses is defined as the date of randomization.

To minimize potential survivor and selection bias, enrollment is restricted to patients within a predefined time window from the most recent AF ablation. Patients must be enrolled within 24 months after ablation, ensuring that randomization occurs during a clinically relevant phase of rhythm stability rather than many years after the index procedure.

In addition, randomization is stratified according to time since the most recent ablation (eg, 12–24 months vs >24 months), and prespecified subgroup analyses will be performed to assess the consistency of treatment effects across different postablation intervals.

By design, OPTIA-AF enrolls a stable CAD population ≥12 months after DES implantation. Patients in the early post-PCI period or with acute coronary syndromes are not included. Complex PCI is permitted and prespecified for stratification at randomization. PCI complexity is defined according to standard contemporary criteria, including multivessel PCI, implantation of 3 or more stents, total stent length of >60 mm, bifurcation PCI requiring 2 stents, left main PCI, or chronic total occlusion intervention. Prespecified subgroup analyses will assess the consistency of treatment effects across PCI complexity strata.

Eligible patients who have maintained stable SR for at least 12 months after AF ablation will be randomized in a 1:1 ratio to either continued NOAC therapy or NOAC discontinuation with transition to SAPT (P2Y12 inhibitor–based strategies), with prespecified safety safeguards in place. This threshold was selected to minimize the influence of transient early recurrences and to ensure a high likelihood of durable rhythm control. The ≥12-month time point also reflects a common clinical milestone at which long-term antithrombotic therapy is reassessed in routine practice, thereby enhancing the translational relevance of the study design. Randomization will be performed using a centralized, Web-based system, with stratification according to DES complexity, CHA_2_DS_2_-VASc score, and renal function, consistent with previous rhythm-guided and antithrombotic trial methodologies.^17^, ^18^, ^19^

In the interventional arm, OAC is discontinued, and SAPT is continued or initiated. In most patients, SAPT represents continuation of background antiplatelet therapy after OAC discontinuation, rather than de novo initiation. SAPT is continued or initiated according to a prespecified framework. SAPT consists of either aspirin or a P2Y12 inhibitor, with P2Y12 inhibitor–based monotherapy recommended as the default strategy in patients with previous DES implantation, unless contraindicated. Aspirin monotherapy may be used in patients with intolerance or contraindication to P2Y12 inhibitors.

To minimize treatment heterogeneity, SAPT selection is guided by predefined clinical criteria rather than unrestricted investigator discretion. The specific antiplatelet agent, dosing, and adherence will be systematically recorded. Subgroup and sensitivity analyses according to SAPT agent type will be performed to assess the consistency of treatment effects, particularly for ischemic and stent-related outcomes.

**Figure 1:**
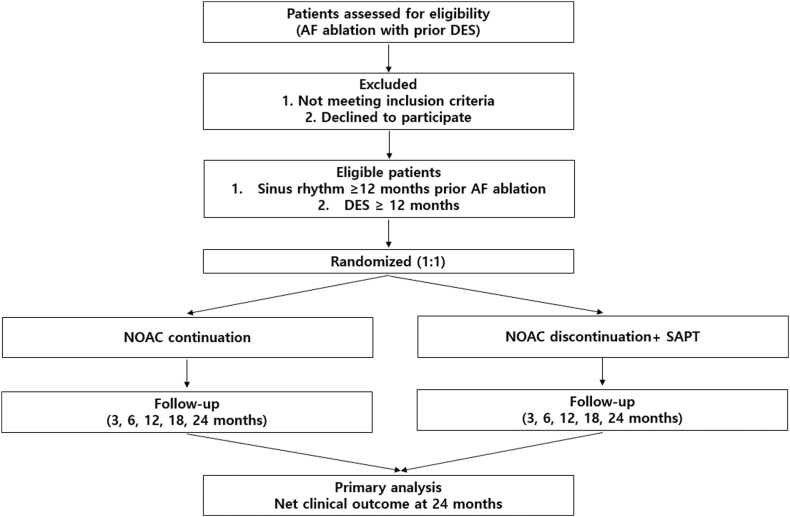
Study flow diagram of the OPTIA-AF trial. This figure illustrates the overall study design and follow-up schema of the OPTIA-AF randomized controlled trial, which evaluates a rhythm-guided antithrombotic strategy after AF ablation in patients with previous DES implantation. Patients with nonvalvular AF who underwent catheter ablation and had a history of DES implantation were screened for eligibility. Eligible patients who maintained durable sinus rhythm for ≥12 months after AF ablation and were ≥12 months removed from the most recent DES implantation were randomized in a 1:1 ratio to either continued NOAC therapy or NOAC discontinuation with initiation of SAPT, with prespecified safety safeguards. Both treatment groups were followed at 3, 6, 12, 18, and 24 months with mandatory rhythm surveillance using electrocardiography and ambulatory or wearable monitoring. The primary end point was the 24-month net clinical outcome, defined as a composite of ischemic stroke, systemic embolism, myocardial infarction, definite or probable stent thrombosis, cardiovascular death, and major bleeding. AF = atrial fibrillation; DES = drug-eluting stent; NOAC = non–vitamin K antagonist oral anticoagulant; OPTIA-AF = Optimal Postablation Therapy for Ischemic and Arrhythmic Risk in Atrial Fibrillation; SAPT = single antiplatelet therapy.

Structured rhythm surveillance using scheduled 12-lead electrocardiography, ambulatory Holter monitoring, and/or wearable digital devices is mandated throughout the study period, as previously described in postablation rhythm monitoring studies.^12^^,^^20^^,^^21^ Patients will be prospectively followed according to their randomized treatment allocation, and clinical events will be systematically ascertained over a 24-month follow-up period. The overall study schema illustrates a rhythm surveillance–driven antithrombotic strategy after successful AF ablation (Figure 1).

#### Primary end point

Primary end points are the 24-month net clinical outcome (ischemic stroke, systemic embolism, definite or probable stent thrombosis, myocardial infarction [MI], cardiovascular death, and major bleeding) between the NOAC maintenance group and the NOAC discontinuation + SAPT group.

To enhance event capture and clinical relevance in the setting of contemporary low postablation thromboembolic risk, the primary composite endpoint includes MI in addition to ischemic stroke, systemic embolism, definite or probable stent thrombosis, cardiovascular death, and major bleeding.

#### Secondary end point

Secondary end points include:

• AF recurrence, defined as any documented episode lasting >30 seconds after randomization
• AF burden assessed by ambulatory or wearable monitoring
• Arrhythmia-related hospitalization
• Repeat catheter ablation

In addition, all individual components of the primary composite end point—including ischemic stroke, systemic embolism, MI, definite or probable stent thrombosis, cardiovascular death, and major bleeding—are prespecified as secondary end points and will be reported separately. Clinically relevant nonmajor bleeding is also included as a secondary safety end point.

As a secondary exploratory analysis, a hierarchical composite endpoint will be evaluated, prioritizing outcomes according to clinical severity in the following order: cardiovascular death, ischemic stroke/systemic embolism, MI, stent thrombosis, and major bleeding.

### End point definition and adjudication

All components of the primary composite end point are defined using standardized clinical criteria and adjudicated centrally. Ischemic stroke is defined as an acute focal neurologic deficit diagnosed by a neurologist and/or requiring hospital admission, with supportive diagnostic evaluation, including neuroimaging when clinically indicated. Routine brain imaging is not mandated, reflecting real-world clinical practice.

Transient ischemic attack is recorded as a separate clinical event but is not included in the primary endpoint. Neurologic syndromes considered stroke mimics, including migraine-related symptoms, seizure-related deficits, or transient postablation neurologic phenomena without objective evidence of cerebral ischemia, are excluded from primary endpoint classification.

Other primary end point components, including MI, definite or probable stent thrombosis, cardiovascular death, and major bleeding, are defined according to established clinical criteria based on hospitalization records, procedural documentation, laboratory findings, and treating physician diagnosis.

All suspected primary end point events are reviewed by an independent clinical event adjudication committee blinded to treatment assignment, to ensure consistency and minimize bias in this open-label trial. For suspected cerebrovascular events, a minimal standardized diagnostic workup is recommended to reduce intercenter variability. Brain imaging with computed tomography and/or magnetic resonance imaging is strongly recommended unless contraindicated. Final classification of ischemic stroke or systemic embolism will be based on integrated clinical assessment, including neurologist evaluation, hospitalization records, and available imaging findings, and will be adjudicated by a blinded clinical event adjudication committee. Transient ischemic attacks and potential stroke mimics will be reviewed and classified according to prespecified criteria.

### Selection of study participants

The selection of study participants is based on predefined inclusion and exclusion criteria, along with clearly specified discontinuation and withdrawal criteria, as summarized in the corresponding tables. Additional safety safeguards are incorporated to ensure participant protection throughout study participation, consistent with previous randomized studies of postablation antithrombotic management.^9^^,^^10^

#### Inclusion criteria

The inclusion criteria (Table 1) are as follows:

• Adults aged ≥19 years
• History of CAD with at least 1 DES implantation
• Nonvalvular AF treated with catheter ablation (initial or repeat procedure)
• Provision of a written informed consent after receiving a full explanation of the study
• Eligibility for study participation and completion of 24-month follow-up as determined by the investigator
• Stable history of AF-related ischemic stroke or transient ischemic attack, provided that they meet all other inclusion criteria and have achieved durable SR

**Table 1:** Inclusion criteria for the OPTIA-AF trial

Inclusion category	Inclusion definition
1. Age	Adults aged ≥19 y at the time of enrollment
2. AF type	Documented nonvalvular AF (paroxysmal or persistent) treated with catheter ablation (index or repeat procedure)
3. Sinus rhythm durability	Maintenance of stable sinus rhythm for ≥12 mo after AF ablation, confirmed by physician documentation and structured rhythm surveillance (ECG, Holter, or wearable monitoring)
4. Coronary artery disease history	Previous PCI with ≥1 DES implantation
5. Timing of DES implantation	≥12 mo elapsed since the most recent DES implantation at the time of enrollment
6. Antithrombotic eligibility	Eligible for either continued NOAC therapy or NOAC discontinuation with SAPT according to the study protocol
7. Informed consent	Provision of a written informed consent after receiving a full explanation of the study during the postablation follow-up period
8. Stable history of AF-related ischemic stroke or TIA	Provided that they meet all other inclusion criteria and have achieved durable sinus rhythm
9. Follow-up feasibility	Willingness and ability to comply with 24-mo follow-up, including clinic visits, rhythm monitoring, and outcome assessment

This table summarizes the eligibility criteria used to identify patients with AF who maintain durable sinus rhythm after catheter ablation and have a history of DES implantation. The criteria define the target study population for evaluation of a rhythm-guided antithrombotic strategy in stable postablation patients.

AF = atrial fibrillation; DES = drug-eluting stent; ECG = electrocardiography; NOAC = non–vitamin K antagonist oral anticoagulant; OPTIA-AF = Optimal Postablation Therapy for Ischemic and Arrhythmic Risk in Atrial Fibrillation; PCI = percutaneous coronary intervention; SAPT = single antiplatelet therapy; TIA = transient ischemic attack.

#### Exclusion criteria

The exclusion criteria (Table 2) are as follows:

• Valvular AF, including mechanical heart valves or moderate-to-severe mitral stenosis
• Acute coronary syndrome or PCI requiring revascularization within the preceding 3 months
• Severe heart failure (left ventricular ejection fraction <30%) or end-stage organ failure
• Intracranial hemorrhage or active major bleeding disorder within the past 6 months
• Malignancy or other conditions associated with a life expectancy of <2 years
• Pregnancy or lactation
• Inability to comply with study procedures or provide an informed consent
• Any condition deemed by the investigator to make study participation inappropriate
• Hypertrophic cardiomyopathy owing to an independent indication for lifelong anticoagulation regardless of rhythm status

**Table 2:** Exclusion criteria for the OPTIA-AF trial

Exclusion category	Exclusion definition
1. Valvular AF	Mechanical prosthetic heart valves or moderate-to-severe mitral stenosis
2. Recent acute coronary syndrome	Acute coronary syndrome or PCI requiring revascularization within 3 mo before enrollment
3. Severe heart failure	LVEF of <30% or advanced heart failure requiring inotropic or mechanical support
4. Major bleeding risk	Intracranial hemorrhage or active major bleeding disorder within the preceding 6 mo
5. End-stage organ disease	End-stage renal, hepatic, or other organ failure limiting life expectancy
6. Malignancy	Active malignancy with an expected life expectancy of <2 y
7. Pregnancy	Pregnant or lactating women or women planning pregnancy during the study period
8. Inability to participate	Inability to provide an informed consent or comply with study procedures
9. Hypertrophic cardiomyopathy	Independent indication for lifelong anticoagulation regardless of rhythm status
10. Investigator discretion	Any condition deemed by the investigator to make participation inappropriate or unsafe

The exclusion criteria were designed to exclude patients with conditions associated with high thromboembolic or bleeding risk, unstable coronary disease, limited life expectancy, or inability to comply with the study protocol, thereby ensuring patient safety and interpretability of clinical outcomes.

ACS = acute coronary syndrome; AF = atrial fibrillation; LVEF = left ventricular ejection fraction; OPTIA-AF = Optimal Postablation Therapy for Ischemic and Arrhythmic Risk in Atrial Fibrillation; PCI = percutaneous coronary intervention.

#### Discontinuation/withdrawal criteria

The discontinuation/withdrawal criteria (Table 3) are as follows:

• Voluntary withdrawal of consent by the participant
• Loss to follow-up for ≥12 months
• Inability to continue participation owing to a serious adverse event
• Discontinuation of the study at the direction of the principal investigator or institutional review board (IRB)

**Table 3:** Discontinuation and withdrawal criteria

Category	Definition
1. Participant withdrawal	Voluntary withdrawal of consent by the participant at any time
2. Loss to follow-up	Inability to contact the participant for ≥12 consecutive mo despite reasonable efforts
3. Serious adverse event	Occurrence of a serious adverse event rendering continued participation clinically inappropriate
4. Protocol or ethical requirement	Study discontinuation directed by the principal investigator, IRB, or regulatory authority

This table outlines predefined criteria for study discontinuation or withdrawal, including participant-initiated withdrawal, loss to follow-up, serious adverse events, or termination required by the principal investigator or IRB.

IRB = institutional review board.

#### SAPT selection is guided by predefined clinical criteria

The predefined clinical criteria that guided SAPT selection (Table 4) are as follows:

• Previous tolerance and ongoing use of aspirin or a P2Y12 inhibitor at enrollment
• Bleeding risk profile (eg, previous major bleeding, HAS-BLED score)
• Coronary ischemic risk and PCI characteristics (eg, stent complexity, history of stent thrombosis)
• A clopidogrel-centered strategy is favored when clinically appropriate, consistent with contemporary AF-PCI evidence.

**Table 4:** SAPT selection is guided by predefined clinical criteria

Category	Clinical criteria and SAPT selection guidance
1. Baseline APT at enrollment	SAPT typically represents continuation of background APT after OAC discontinuation rather than de novo initiation.
2. Default SAPT strategy	P2Y12 inhibitor–based monotherapy (preferably clopidogrel) is recommended as the default SAPT strategy in patients with previous DES implantation.
3. Aspirin use	Aspirin monotherapy may be selected in patients with contraindication or intolerance to P2Y12 inhibitors.
4. Bleeding risk profile	History of major bleeding, high HAS-BLED score, or frailty favors SAPT rather than combined antithrombotic therapy; the choice of agent should minimize bleeding risk.
5. Coronary ischemic risk and PCI characteristics	Features such as complex PCI (eg, ≥3 stents, total stent length >60 mm, bifurcation PCI with 2 stents, left main PCI, chronic total occlusion intervention) favor continuation of SAPT with a P2Y12 inhibitor rather than aspirin alone.
6. History of stent-related events	Previous stent thrombosis or recurrent ischemic events favor clopidogrel-based SAPT when clinically appropriate.
7. Drug tolerance and adherence	Previous tolerance, adherence, and absence of adverse reactions to aspirin or P2Y12 inhibitors are considered in SAPT selection.
8. Investigator discretion within protocol framework	Final SAPT selection is made within the predefined criteria above; unrestricted investigator discretion is not permitted. All SAPT choices are prospectively documented.

APT selection criteria were prespecified to minimize treatment heterogeneity and allow mechanistic interpretation of ischemic and stent-related outcomes. This table summarizes the predefined clinical criteria used to guide the SAPT selection after discontinuation of oral anticoagulation in the OPTIA-AF trial. The framework is designed to minimize treatment heterogeneity while reflecting contemporary coronary practice and patient-specific ischemic and bleeding risk profiles.

APT = antiplatelet therapy; DAPT = dual antiplatelet therapy; DES = drug-eluting stent; OAC = oral anticoagulation; OPTIA-AF = Optimal Postablation Therapy for Ischemic and Arrhythmic Risk in Atrial Fibrillation; PCI = percutaneous coronary intervention; SAPT = single antiplatelet therapy.

### Safety safeguards

Patient safety is prioritized through continuous surveillance for adverse events, including systematic monitoring for serious adverse events and major bleeding, in accordance with IRB-approved protocols and contemporary antithrombotic trial standards.^17^, ^18^, ^19^

In the event of documented AF recurrence lasting >30 seconds after randomization or the occurrence of clinically significant thromboembolic risk, resumption of OAC is prespecified and strongly recommended, irrespective of randomized treatment allocation, to ensure arrhythmia-related thromboembolic safety.^12^

AF recurrence is defined as any documented episode lasting >30 seconds after randomization and serves as a protocol-defined safety trigger for consideration of OAC resumption. This threshold was selected to prioritize patient safety and is consistent with conventional definitions used in postablation follow-up. To account for emerging evidence that longer AF episodes and cumulative AF burden are more closely associated with thromboembolic risk, complementary secondary and exploratory analyses will evaluate AF recurrence using alternative thresholds (eg, episode duration ≥5–6 minutes) and quantitative AF burden assessed by ambulatory or wearable monitoring. These analyses are intended to contextualize crossover patterns and treatment effects beyond the binary >30-second definition. Secondary and exploratory analyses based on longer AF episode duration and cumulative AF burden are intended to be hypothesis generating and contextual in nature. Given the protocol-defined recommendation to resume OAC after any AF recurrence lasting >30 seconds, these analyses are not designed to support independent treatment decisions but rather to inform interpretation of rhythm dynamics and crossover patterns.

Management of AF recurrence during follow-up, including repeat catheter ablation, antiarrhythmic drug therapy, or electrical cardioversion, will be recorded prospectively. These interventions will be accounted for in prespecified sensitivity analyses to contextualize their potential influence on subsequent thromboembolic risk.

### Follow-up and study data collection

This study is a prospective, multicenter, randomized controlled trial. Patients who visited the hospital within 3 months after AF ablation were included. After assessing the duration of DES, SR stability, and risk of CAD/PCI, eligible patients will be randomized in a 1:1 fashion to either continued NOAC therapy or NOAC discontinuation with SAPT using a centralized, Web-based randomization system. Patients will be prospectively followed according to their randomized treatment allocation.

#### Baseline assessment

• Demographic information, underlying disease, CHA_2_DS_2_-VASc, and HAS-BLED scores
• Timing of DES implantation, lesion location, complicated PCI, and number of stents
• Echocardiography (left ventricular ejection fraction, left atrial size, valvular disease, etc)
• AF ablation details and early AF recurrence

#### Follow-up visits and observation items

• Follow-up visits: outpatient visits or telephone follow-up at 3, 6, 12, 18, and 24 months after enrollment
• Event ascertainment: ischemic stroke/systemic embolism, stent thrombosis, cardiovascular death, major bleeding, AF recurrence, readmission, and re-PCI
• Rhythm assessment: 12-lead electrocardiogram, and, if necessary, assessment of AF burden through Holter or remote monitoring.^12^^,^^20^ Rhythm monitoring is a mandatory component of the study protocol. All participants undergo scheduled 12-lead electrocardiography at each follow-up visit, supplemented by ambulatory Holter monitoring and/or wearable rhythm monitoring devices to quantify AF recurrence and AF burden throughout the 24-month follow-up period.

### Rhythm monitoring strategy

Rhythm surveillance in OPTIA-AF is conducted using structured but pragmatic monitoring strategies. All participants undergo scheduled 12-lead electrocardiography at follow-up visits. In addition, 24-hour Holter monitoring is recommended, and wearable Holter patches or digital rhythm monitoring devices may be used when available, according to local center resources and practice patterns.

Continuous wearable monitoring is not mandated for all participants. This flexible approach was intentionally adopted to reflect real-world clinical practice and to ensure feasibility and generalizability across centers with varying access to monitoring technologies. Rhythm monitoring data are used to document AF recurrence and to guide protocol-defined safety measures, including resumption of OAC when indicated.

Rhythm assessment in OPTIA-AF follows a standardized minimum surveillance framework for all participants. Scheduled 12-lead electrocardiography is performed at each follow-up visit. In addition, ambulatory rhythm monitoring of at least 24 hours’ duration, using either conventional Holter monitoring or an equivalent wearable Holter patch device, is mandated at prespecified follow-up intervals.

Continuous or extended wearable rhythm monitoring beyond this minimum requirement may be performed according to local center practice and resource availability but is not required for protocol adherence. This approach was intentionally adopted to ensure consistent ascertainment of AF recurrence across centers, while preserving feasibility and external validity.

Adherence to rhythm monitoring and completeness of rhythm data will be prospectively documented. Prespecified procedures for handling missing or incomplete rhythm data are incorporated into the statistical analysis plan to facilitate robust and interpretable assessment of rhythm durability and recurrence.

### Sample size calculation

The primary endpoint is the 24-month net clinical outcome. Event-rate assumptions were informed by contemporary randomized trials evaluating anticoagulation strategies after AF ablation and by AF-PCI literature, in which reported 24-month net clinical event rates generally range from approximately 3%–7%.^9^^,^^10^^,^^14^, ^15^, ^16^^,^^22^

For the primary noninferiority analysis, a conservative 24-month cumulative incidence of 5.0% is assumed for the continued NOAC group. Noninferiority will be tested using an absolute risk-difference margin (Δ) of 4.0 percentage points, with a 1-sided α of 0.025, 80% power, and 1:1 randomization, consistent with previous noninferiority designs in antithrombotic trials.^23^

Allowing for up to 10% loss to follow-up, the target enrollment for OPTIA-AF is set at 1000 patients (500 per group). Sensitivity analyses across a range of assumed event rates and noninferiority margins were performed to confirm the robustness of the selected sample size assumptions and are presented in the Supplemental tables.

Although contemporary rhythm-guided anticoagulation trials are challenged by low absolute event rates, OPTIA-AF was specifically designed to address this limitation by broadening the primary endpoint to include MI and enrolling a population with residual coronary ischemic risk. This strategy seeks to avoid an underpowered, noninformative result while preserving patient safety and clinical relevance.

### Study organization and feasibility

OPTIA-AF is designed as a multicenter randomized controlled trial involving approximately 10–15 participating centers in the Republic of Korea. Participating centers represent a broad spectrum of procedural volumes, with annual AF ablation case numbers ranging from approximately 50–300 procedures per center, reflecting real-world clinical practice.

Based on these volumes and anticipated eligibility rates, each center is expected to enroll approximately 30–150 patients during the enrollment period, yielding a total target sample size of approximately 1000 participants. Enrollment is planned over 24 months, followed by 24 months of prospective clinical follow-up for all participants.

A 1-year preparatory phase before enrollment is planned to allow for regulatory approvals, site initiation, investigator training, and protocol harmonization. Accordingly, the total projected study duration is approximately 5 years, encompassing preparation, enrollment, follow-up, and final analysis.

### Interim analysis and stopping considerations

A prespecified interim analysis is planned after 1 year of follow-up to evaluate safety and event trends. Early termination of the trial may be considered after this interim analysis if the primary endpoint is met or if there is a substantial and clinically meaningful excess of primary endpoint events in the experimental group that clearly favors the control group. Decisions regarding early termination will be guided by the totality of safety and efficacy data and overseen according to predefined monitoring procedures.

### Subgroup analyses

Prespecified subgroup analyses will be performed to explore the consistency of treatment effects across clinically relevant strata. Subgroups of interest include patient characteristics (age [<65 vs ≥65 years], sex, baseline CHA_2_DS_2_-VASc score [≤2 vs ≥3], renal function), AF-related factors (AF type, initial vs repeat ablation, time since last ablation), and PCI-related parameters (time since DES implantation, number of stents, and presence of complex PCI features).

In addition, rhythm-related subgroups, including documented AF recurrence and AF burden during follow-up, will be evaluated. Subgroup analyses will be conducted using interaction testing within Cox proportional hazards models. All subgroup analyses are prespecified, exploratory in nature, and intended to be hypothesis generating rather than definitive.

### Statistical analysis

Continuous variables are presented as means ± standard deviations or medians (interquartile ranges), as appropriate, and compared using the independent-samples *t* test or the Mann–Whitney U test. Categorical variables are expressed as counts and percentages and compared using the χ^2^ test or Fisher’s exact test, as appropriate.

Time-to-event outcomes are analyzed using Kaplan–Meier estimates, with between-group comparisons performed using the log-rank test. Hazard ratios with 95% confidence intervals are estimated using Cox proportional hazards models, adjusted for prespecified clinically relevant covariates, including age, sex, CHA_2_DS_2_-VASc score, and DES complexity. The proportional hazards assumption is assessed using Schoenfeld residuals.

The primary analysis is conducted according to the intention-to-treat principle. For the noninferiority assessment of the primary composite endpoint, the absolute risk difference between groups and its corresponding 1-sided 97.5% confidence interval are estimated. Noninferiority is concluded if the upper bound of the confidence interval does not exceed the prespecified noninferiority margin. A per-protocol analysis is additionally performed as a sensitivity analysis to assess the robustness of the primary findings.

In addition to the primary noninferiority analysis of the net clinical outcome, all individual components of the composite endpoint will be reported separately. Prespecified secondary analyses will include component-wise comparisons and hierarchical analyses to facilitate clinically meaningful interpretation in the event that ischemic and bleeding components demonstrate divergent directional effects.

In the event of treatment crossover, particularly resumption of OAC owing to documented AF recurrence, the primary analysis will follow the intention-to-treat principle. To assess the robustness of the findings and the impact of treatment crossover on treatment separation, prespecified per-protocol and as-treated sensitivity analyses will also be performed. The frequency, timing, and reasons for crossover will be systematically recorded, and treatment separation will be explicitly reported. These complementary analyses are intended to facilitate interpretation of the noninferiority results, particularly if crossover rates exceed anticipated thresholds.

Secondary endpoints, including AF recurrence (defined as any documented episode lasting >30 seconds after randomization), AF burden, arrhythmia-related hospitalization, and repeat catheter ablation, are analyzed using appropriate time-to-event or categorical methods. Given the exploratory nature of secondary endpoints, no formal adjustment for multiple comparisons is applied.

Hierarchical composite analyses will be performed as secondary analyses to facilitate clinically meaningful interpretation of outcomes with differing clinical importance.

Sensitivity analyses will be performed using alternative AF recurrence definitions based on episode duration and AF burden to assess the robustness of the primary findings and the impact of rhythm thresholds on treatment crossover.

All statistical tests are 2 sided unless otherwise specified. *P* < .05 is considered statistically significant for secondary and exploratory analyses. All analyses are performed using IBM SPSS Statistics version 26.0 (IBM Corp, Armonk, NY).

### Ethical approval

This manuscript describes the design and rationale of a proposed randomized clinical trial. Ethical approval will be obtained from the IRBs of all participating centers before patient enrollment, and a written informed consent will be obtained from all participants. The study will be conducted in accordance with the principles of the Declaration of Helsinki and applicable regulatory requirements.^24^^,^^25^ The trial will be registered in a publicly accessible clinical trial registry before the enrollment of the first patient. An independent data and safety monitoring process will be implemented to ensure ongoing participant safety throughout the trial.

## Discussion

### Principal concept and electrophysiological perspective

OPTIA-AF was designed from an electrophysiological perspective in which durable SR after catheter ablation is regarded as a clinically actionable variable rather than a static historical diagnosis of AF. By focusing on patients who maintain sustained SR for at least 12 months after ablation and have stable CAD with previous DES implantation, this trial directly addresses a clinically important gap in long-term antithrombotic decision making at the intersection of electrophysiology and coronary disease management.

In contemporary practice, antithrombotic therapy after AF ablation is largely determined by baseline stroke risk stratification, irrespective of long-term rhythm outcomes. However, accumulating evidence suggests that successful ablation with durable rhythm control substantially reduces AF burden and AF-related thromboembolic risk.^5^, ^6^, ^7^^,^^26^ OPTIA-AF challenges the prevailing paradigm by evaluating whether sustained rhythm control can be integrated into antithrombotic decision making without compromising overall clinical safety.

### Comparison with previous studies and guidelines

Previous studies have explored the feasibility of discontinuing OAC after successful AF ablation in selected patients, particularly those with low CHA_2_DS_2_-VASc scores.^8^, ^9^, ^10^ However, these investigations generally excluded patients with significant CAD or did not specifically consider populations requiring ongoing antiplatelet therapy, limiting their applicability to patients with previous DES implantation.

Current guideline recommendations for patients with AF and previous PCI generally favor OAC monotherapy beyond 12 months after DES implantation to reduce bleeding risk.^11^, ^12^, ^13^ Although supported by randomized evidence in stable coronary disease, these recommendations implicitly assume persistent AF-related thromboembolic risk and do not adequately address patients in whom AF recurrence has been effectively suppressed by durable SR after ablation. OPTIA-AF contextualizes these guidelines by prospectively evaluating a population in which rhythm stability is confirmed through structured surveillance and residual coronary ischemic risk persists.

In addition, real-world data from higher-risk settings provide further support for de-escalation strategies over time. Registry studies of patients with AF and acute coronary syndromes undergoing PCI demonstrate that stepwise simplification of antithrombotic therapy is common in clinical practice and can be associated with acceptable long-term net clinical outcomes.^14^, ^15^, ^16^, ^22^ Compared with these populations, OPTIA-AF targets a lower-risk cohort characterized by stable coronary disease and durable postablation SR.

### Mechanistic rationale

The mechanistic rationale underlying OPTIA-AF is grounded in the divergent pathophysiology of thromboembolic events in AF and ischemic events after DES implantation. AF-related thromboembolism arises primarily from atrial rhythm instability, atrial stasis, and left atrial appendage thrombosis.^14^, ^15^, ^16^ In contrast, late ischemic events after PCI are predominantly platelet mediated and related to vascular healing and local stent pathology.^27^, ^28^, ^29^, ^30^

In patients who maintain durable SR after catheter ablation, atrial arrhythmogenicity and stasis are substantially reduced, potentially attenuating the incremental benefit of continued systemic anticoagulation. In this context, antiplatelet therapy may more directly target the dominant residual ischemic risk related to CAD. OPTIA-AF was specifically designed to test this mechanistic hypothesis within a randomized framework while incorporating mandatory rhythm surveillance to ensure accurate assessment of rhythm stability.

Evidence from randomized trials in stable coronary disease also supports a risk-adapted de-escalation framework. In a contemporary meta-analysis of randomized trials enrolling patients with AF and stable CAD, OAC monotherapy was not associated with a higher risk of ischemic events than OAC plus SAPT, while significantly reducing major bleeding.^31^ These findings suggest that, beyond the acute phase of PCI, incremental intensification of antithrombotic therapy may yield limited ischemic benefit at the expense of bleeding. In OPTIA-AF, durable SR after ablation may further attenuate AF-related thromboembolic risk, shifting the dominant residual risk toward platelet-mediated coronary events. Within this context, evaluating a rhythm-guided transition from NOAC to SAPT is clinically and mechanistically aligned with the broader randomized evidence base favoring simplified regimens in stable CAD, while directly addressing the unique management challenge of patients who require ongoing coronary protection yet may no longer derive net benefit from continued systemic anticoagulation.

Although contemporary rhythm-guided anticoagulation trials are challenged by low absolute event rates, OPTIA-AF was specifically designed to address this limitation by broadening the primary endpoint to include MI and by enrolling a population with residual coronary ischemic risk. This strategy seeks to avoid an underpowered, noninformative result while preserving patient safety and clinical relevance.

Although a >30-second definition of AF recurrence remains standard for ablation outcome assessment, accumulating evidence from device-detected AF studies suggests that stroke risk correlates more strongly with longer episode duration and cumulative AF burden. OPTIA-AF addresses this evolving understanding by prespecifying burden-based and longer-duration AF analyses, allowing interpretation of trial results within both traditional safety-oriented and biologically informed rhythm frameworks. Importantly, analyses based on longer AF episode duration and cumulative AF burden should be interpreted within the context of the protocol-mandated safety framework. Because OAC is recommended to be resumed after any documented AF recurrence exceeding 30 seconds, subsequent rhythm-based subgroup analyses are exploratory and primarily intended to provide mechanistic and contextual insight rather than definitive evidence to guide clinical decision making.

### Antiplatelet selection and contemporary evidence

Although earlier studies evaluating aspirin-based strategies have raised concerns regarding ischemic protection after anticoagulation withdrawal, contemporary evidence suggests that aspirin may no longer represent the optimal long-term antiplatelet agent in stable CAD. Recent randomized trials and large pooled analyses have demonstrated that clopidogrel monotherapy is associated with a lower risk of major adverse cardiovascular events than aspirin, with similar rates of major bleeding, in patients with established coronary disease. These findings have contributed to a paradigm shift away from aspirin-centered maintenance therapy toward P2Y12 inhibitor–based strategies in selected chronic coronary settings.^32^

Consistent with this evolving evidence, major AF-PCI trials and guideline recommendations now preferentially support P2Y12 inhibitor–based regimens, most commonly clopidogrel, while limiting aspirin exposure to the early post-PCI period. In this context, the numerically higher ischemic event rates observed with aspirin in some previous studies, including OCEAN, may reflect limitations of aspirin rather than an inherent failure of antiplatelet-based strategies per se.

Therefore, OPTIA-AF evaluates a clopidogrel-centered SAPT strategy, rather than aspirin alone, in patients with durable SR after AF ablation and remote DES implantation. This approach aligns with contemporary coronary practice and provides a more biologically and clinically relevant framework for assessing whether rhythm-guided de-escalation from anticoagulation can be achieved without compromising ischemic safety.

### Clinical implications

If noninferiority is demonstrated, OPTIA-AF has the potential to inform a more individualized, risk-based approach to long-term antithrombotic therapy after AF ablation. Rather than applying a uniform anticoagulation strategy to all patients with a history of AF, the trial evaluates whether antithrombotic therapy can be tailored according to rhythm durability, residual coronary ischemic risk, and bleeding propensity.^33^

Importantly, OPTIA-AF does not propose universal discontinuation of anticoagulation after ablation, nor does it suggest that SAPT can replace anticoagulation in all patients. Instead, it seeks to identify a clinically distinct subgroup in whom a rhythm-guided transition to antiplatelet-centered therapy may offer a favorable balance between ischemic protection and bleeding risk, consistent with contemporary efforts toward precision medicine in cardiovascular care.^34^^,^^35^

Importantly, interpretation of the net clinical outcome will be guided by prespecified component-wise and hierarchical analyses. In scenarios where reductions in bleeding events are offset by increases in ischemic events, or vice versa, individual endpoint components—including MI, stroke, and major bleeding—will be examined separately to inform the clinical acceptability of the observed trade-offs, rather than relying solely on the composite outcome.

Although protocol-mandated resumption of anticoagulation in response to AF recurrence may attenuate treatment separation, this approach reflects real-world clinical practice and prioritizes patient safety. Prespecified sensitivity analyses will be essential to contextualize the primary intention-to-treat results and to inform the interpretation of the findings under varying degrees of crossover.

### External validity and pragmatic considerations

Although OPTIA-AF incorporates structured rhythm surveillance, the monitoring strategy is designed to remain pragmatic and reflective of contemporary clinical practice. The trial does not depend on universal use of wearable or continuous monitoring devices; rather, it evaluates the clinical relevance of rhythm status assessed using commonly available tools such as electrocardiography and ambulatory Holter monitoring.

By allowing center-specific monitoring approaches, OPTIA-AF seeks to balance biological rigor with external validity. The findings are therefore intended to inform clinical decision making in a wide range of cardiology practice settings, including those in which advanced wearable monitoring technologies are not routinely available.

### Future directions

Future studies should focus on external validation of rhythm-guided antithrombotic strategies in larger and more diverse populations. Incorporation of continuous rhythm monitoring technologies and biomarkers of atrial remodeling and endothelial dysfunction may further refine patient selection and risk stratification.^20^^,^^21^ Future studies incorporating continuous rhythm monitoring and formal cost-effectiveness analyses may further refine patient selection and clarify the real-world applicability of rhythm-guided antithrombotic strategies in routine electrophysiology practice.

### Limitations

Several limitations warrant consideration. First, the generalizability of OPTIA-AF may be constrained by the requirement for durable SR confirmed through systematic rhythm monitoring. Second, although the study is powered to assess net clinical outcomes, it may be underpowered to detect modest differences in individual components of the composite endpoint. Third, the open-label nature of antithrombotic therapy may introduce bias, although clinical events are adjudicated in a blinded manner. Finally, although the selected noninferiority margin reflects a balance between clinical relevance and feasibility, smaller differences between strategies cannot be excluded.^23^

Importantly, the PCI population studied represents a stable, late post–DES cohort. Accordingly, the findings of OPTIA-AF should not be extrapolated to patients in the early post-PCI period or those with acute coronary syndromes. In addition, consistent with contemporary PCI literature, selected patients with complex or high-ischemic-risk PCI may warrant prolonged or intensified antiplatelet therapy beyond 12 months. Therefore, the applicability of a rhythm-guided SAPT strategy should be interpreted in light of PCI complexity and residual coronary ischemic risk. This approach aligns with contemporary PCI literature in which prolonged or intensified antiplatelet therapy beyond 12 months is selectively considered in patients with complex or high-ischemic-risk PCI, rather than uniformly applied.

## Conclusion

The OPTIA-AF trial is designed to address a clinically important and unresolved question in the long-term management of patients with AF who maintain durable SR after catheter ablation and have a history of DES implantation. By integrating objective rhythm durability with residual coronary ischemic risk and bleeding propensity, this trial evaluates a rhythm-guided antithrombotic strategy that transitions selected patients from NOAC to SAPT compared with continued anticoagulation. OPTIA-AF aims to generate randomized evidence to inform a more individualized, risk-adapted approach to antithrombotic therapy after AF ablation in patients with concomitant CAD.

## Disclosures

The authors have no conflicts of interest to disclose.

## Footnotes

Appendix Supplementary data associated with this article can be found in the online version at https://doi.org/10.1016/j.hroo.2026.02.002.
