# Supplementary material for: OPTIA-AF trial: A randomized study of rhythm-guided antithrombotic strategy after atrial fibrillation ablation in patients with prior drug-eluting stent implantation
Source: Heart Rhythm O2. 2026 Feb 9;7(4):774–85. doi: 10.1016/j.hroo.2026.02.002 (PMC13107038; doi:10.1016/j.hroo.2026.02.002)
Supplement: Supplementary Tables [file mmc1.docx]

**Supplementary Table S1. Assumed Event Rates for Long-Term Net Clinical Outcomes in Contemporary AF and AF–PCI Studies**

This table summarizes representative ranges of net clinical event rates reported in contemporary studies evaluating long-term antithrombotic outcomes in patients with atrial fibrillation (AF), including post–catheter ablation cohorts and populations with concomitant coronary artery disease (CAD) or prior percutaneous coronary intervention (PCI). Across these studies, reported 24-month net clinical event rates generally range from approximately 3% to 7%, depending on study population, background therapy, and endpoint definitions.

Based on these data, a **conservative cumulative 24-month event rate of 5.0%** was assumed for the continued non–vitamin K antagonist oral anticoagulant (NOAC) group in the OPTIA-AF trial **solely for the purpose of sample size estimation**.

**Abbreviations**: AF, atrial fibrillation; CAD, coronary artery disease; PCI, percutaneous coronary intervention; SE, systemic embolism; NOAC, non–vitamin K antagonist oral anticoagulant.

| **Study / Population** | **Follow-up** | **Outcome Type** | **Event Rate** |
| --- | --- | --- | --- |
| AF ablation cohorts (durable sinus rhythm) | 12–24 months | Stroke / SE, major bleeding | 2–4% |
| AF + CAD/PCI long-term therapy studies | 24 months | Net clinical composite | 4–7% |
| Contemporary AF-PCI randomized trials | ≥12 months | Ischemic + bleeding composite | 5–8% |
| **OPTIA-AF assumed control group rate** | **24 months** | **Net clinical composite** | **5.0%** |

**Supplementary Table S2. Sensitivity Analysis of Sample Size Requirements Under Varying Assumptions**

This table presents sensitivity analyses of the required total sample size under varying assumptions of control-group event rates and non-inferiority margins. Sample size estimates are based on a non-inferiority design using an absolute risk-difference approach, assuming equal event rates between treatment groups, a one-sided alpha level of 0.025, and 80% statistical power.

The column corresponding to Δ = 4.0 percentage points reflects the primary design assumption of the OPTIA-AF trial, while smaller margins are shown for contextual sensitivity analyses.

**Abbreviations:** Δ, absolute non-inferiority margin (percentage-point difference); pC, assumed cumulative event rate in the control group (continued NOAC therapy).

| **Assumed Event Rate**  **(pC)** | **Δ = 1.5%p** | **Δ = 2.0%p** | **Δ = 2.5%p** | **Δ = 4.0%p**  **(OPTIA-AF design)** |
| --- | --- | --- | --- | --- |
| 3% | ~1700 | ~950 | ~610 | ~610 |
| 5% | ~1250 | ~860 | ~560 | ~560 |
| 7% | ~1050 | ~740 | ~490 | ~490 |

Values represent approximate total sample sizes required for both treatment groups combined.
